# Validation of the WHO-defined 20% circulating blasts threshold for diagnosis of leukemic transformation in primary myelofibrosis

**DOI:** 10.1038/s41408-018-0095-2

**Published:** 2018-06-11

**Authors:** Mythri Mudireddy, Naseema Gangat, Curtis A. Hanson, Rhett P. Ketterling, Animesh Pardanani, Ayalew Tefferi

**Affiliations:** 1 Department of Internal Medicine and Laboratory Medicine, Division of Hematology, Mayo Clinic, Rochester, MN USA; 2 Department of Internal Medicine and Laboratory Medicine, Division of Hematopathology, Mayo Clinic, Rochester, MN USA; 3 Department of Internal Medicine and Laboratory Medicine, Division of Laboratory Genetics and Genomics, Mayo Clinic, Rochester, MN USA

Leukemic transformation is the most dreaded complication in patients with myeloproliferative neoplasms (MPN); overall risk is estimated at 14% for primary myelofibrosis (PMF), 7% for polycythemia vera (PV) and 4% for essential thrombocythemia (ET)^1^. The dismal prognosis of blast phase MPN (MPN-BP) was recently underlined in a large Mayo Clinic study of 248 informative patients and further validated in a separate cohort of 162 cases recruited from multiple Italian institutions^2^. Current treatment approaches for MPN-BP, including allogeneic hematopoietic stem cell transplant (HCT), are largely ineffective in securing long-term survival^2^. On the other hand, HCT has been shown to produce long-term remissions in ~50% of patients with myelofibrosis, if undertaken before blast transformation^3^. At present, diagnosis of MPN-BP employs the World Health Organization (WHO)-defined criteria for acute myeloid leukemia (AML): presence of ≥20% blasts either in the bone marrow (BM) or in the peripheral blood (PB)^4^. In other words, neither scenario requires compartmental concordance. However, it is currently not clear if this 20% WHO threshold for circulating blasts is valid in the context of primary myelofibrosis (PMF), especially in terms of its prognostic equivalency to that of BM blast percentage-defined blast phase PMF (PMF-BP) and its distinction from accelerated phase disease (PMF-AP), operationally defined by the presence of 10–19% circulating blasts^5^.

The current study was approved by the Mayo Clinic institutional review board. Diagnosis of PMF and PMF-BP were according to WHO criteria^4^. Designation of unfavorable karyotype was according to previously published criteria^6^. Study patients were recruited from institutional databases of chronic phase or blast phase PMF. As per the study design, phenotypic and prognostic comparisons considered four distinct categories of PMF patients: (i) PMF with 5–9% circulating blasts, (ii) PMF-AP with 10–19% circulating blasts, (iii) PMF-BP with ≥20% BM blasts, regardless of PB blast percentage (i.e., BM-defined PMF-BP), and (iv) PMF-BP with ≥20% PB blasts but <20% BM blasts (i.e., PB-defined PMF-BP). Statistical analyses for chronic phase PMF considered clinical and laboratory data collected at the time of documented PB blast count of ≥5% and for PMF-BP, date of leukemic transformation. Survival was computed from the date of either leukemic transformation (for PMF-BP) or documentation of the increased blast threshold for PMF-AP and PMF with 5–9% circulating blasts. Non-parametric statistics was used to determine significance of differences among groups, in the distribution of continuous or nominal variables. The Stat View (SAS Institute, Cary, NC, USA) statistical package was used for all calculations.

The total number of study patients was144 and included 41 patients with chronic phase PMF and 5–19% circulating blasts and 103 with PMF-BP (Table 1); the 41 patients with chronic phase PMF included 28 patients with 5–9% circulating blasts and 13 patients with 10–19% circulating blasts (i.e., PMF-AP); the 103 patients with PMF-BP included 71 patients in whom diagnosis was confirmed by the demonstration of ≥20% BM blasts and 32 patients in whom BM examination revealed <20% blasts but diagnosis of PMF-BP was based on the presence of ≥20% PB blasts. These four operational groups of patients with PMF displayed similar age (*p* = 0.6) and gender (*p* = 0.6) distribution and were also similar in their need for red cell transfusions (*p* = 0.4), hemoglobin level (*p* = 0.9), leukocyte count (*p* = 0.9), and driver mutational status (*p* = 0.3), including the incidence of type 1/like *CALR* mutations (11% vs 15% vs 17% vs 19%, respectively) (Table 1). On the other hand, platelet counts were significantly lower in both BM-defined (median 67 × 10^9^/l) and PB-defined (median 79 × 10^9^/l) PMF-BP, compared to those seen in PMF patients with 5–9% circulating blasts (median 157 × 10^9^/l) or PMF-AP (median 139 × 10^9^/l) (*p* = 0.004). Significant differences were also noted for karyotype, as detailed in Table 1, and the incidences of abnormal (*p* = 0.02) and unfavorable (*p* = 0.03) karyotype were the highest in patients with PB-defined PMF-BP and lowest in PMF with 5–9% circulating blasts.

**Table 1:** Comparison of clinical and laboratory parameters between “blast phase primary myelofibrosis (PMF-BP)” and “chronic phase primary myelofibrosis with 5–19% circulating blasts”

Variables	All patients (*n* = 144)	PMF-BP with ≥20% BM blasts (*n* = 71)	PMF-BP with<20% BM blasts but ≥20% PB blasts (*n* = 32)	PMF-AP with 10–19% PB blasts (*n* = 13)	PMF with 5–9% PB blasts (*n* = 28)	*P* value
Age in years; median (range)	68 (43–89)	68 (44–87)	66 (44–84)	64 (46–80)	69 (43–89)	0.6
Age >65 years; *n* (%)	81 (56%)	41 (58%)	16 (50%)	6 (46%)	18 (64%)	0.6
Sex (male); *n* (%)	96 (67%)	49 (69%)	23 (72%)	7 (54%)	17 (61%)	0.6
Transfusion dependent; *n* (%)	68 (48%)	31 (44%)	13 (42%)	7 (54%)	17 (61%)	0.4
Hemoglobin, g/dl; median (range)	9.3(6.1–13.7)	9.1 (6.1–13.7)	8.9 (6.3–11.2)	10 (7.0–11.6)	9.7 (6.6–13.5)	0.88
Platelets, ×10^9^/L; median (range)	79 (4–984)	67 (4–568)	79 (6–670)	139 (24–885)	157 (14–984)	**0.004**
Leukocytes, ×10^9^/L; median (range)	21 (0.5–219)	22.3 (0.5–208.4)	19.5 (3.0–139.5)	21.5 (2.1–75)	20.1 (1.8–219)	0.9
Karyotype “N” evaluable = 119 (83%)						**0.03**
Favorable; *n* (%)	64 (54%)	31 (51%)	7 (39%)	5 (38%)	21 (78%)	
Unfavorable; *n* (%)	55(46%)	30 (49%) (N evaluable = 61)	11 (61%)(N evaluable = 18)	8 (62%)	6 (22%) (N evaluable = 27)	
Karyotype “N” evaluable = 119 (83%)						**0.02**
Normal; *n* (%)	35 (29%)	16 (26%)	2 (11%)	3 (23%)	14 (52%)	
Abnormal; *n* (%)	84 (71%)	45 (74%) (N evaluable = 61)	16 (89%) (N evaluable = 18)	10 (77%)	13 (48%) (N evaluable = 27)	
Driver mutation status “N” evaluable = 113 (78%)						0.3
*JAK2*; *n* (%)	67 (59%)	32 (67%)	12 (50%)	8 (62%)	15 (53%)	
*CALR* Type 1/like; *n* (%)	18 (16%)	9 (19%)	4 (17%	2 (15%)	3 (11%)	
*CALR* Type 2/like; *n* (%)	9 (8%)	1 (2%)	2 (8%)	3 (23%)	3 (11%)	
*MPL*; *n* (%)	6 (5%)	2 (4%)	1 (4%)	0 (0%)	3 (11%)	
Triple-negative; *n* (%)	13 (12%)	4 (8%)(N evaluable = 48)	5 (21%)(N evaluable = 24)	0 (0%)	4 (14%)	

The values in bold indicate a significant *p*-value < 0.05

*BM* bone marrow, *PB* peripheral blood, *PMF* primary myelofibrosis, *PMF-BP* blast phase PMF, *PMF-AP* accelerated phase PMF with 10–19% circulating blasts, *JAK2* Janus kinase 2, *CALR* Calreticulin, *MPL* MPL proto-oncogene

**Fig. 1:**
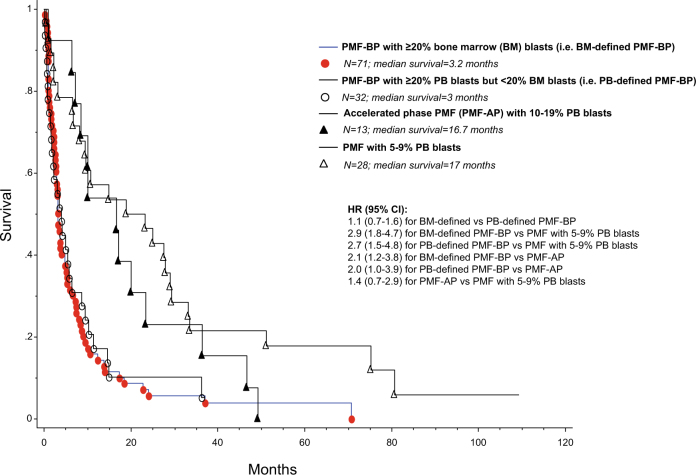
Overall survival of 144 patients with either “chronic phase primary myelofibrosis with 5–19% peripheral blood (PB) blasts” or “blast phase primary myelofibrosis (PMF-BP)”

Most importantly, survival data of BM-defined PMF-BP (≥20% BM blasts) were indistinguishable from those of PB-defined PMF-BP (<20% BM blasts but ≥20% PB blasts) (HR 1.1; 95% CI 0.7–1.6; *p* = 0.8; Fig. 1), whereas survival in both instances was significantly shorter than that of patients with chronic phase PMF with 5–19% circulating blasts: HRs (95% CI) were 2.6 (1.7–3.8) for PMF-BP (both BM- and PB-defined combined) vs PMF with 5–19% circulating blasts; 2.9 (1.8–4.7) for BM-defined PMF-BP vs PMF with 5–9% circulating blasts; 2.7 (1.5–4.8) for PB-defined PMF-BP vs PMF with 5–9% circulating blasts; 2.1 (1.2–3.8) for BM-defined PMF-BP vs PMF-AP; and 2.0 (1.0–3.9) for PB-defined PMF-BP vs PMF-AP (Fig. 1). Survival was not significantly different between PMF-AP and PMF with 5–9% circulating blasts (HR 1.4, 95% CI 0.7–2.9; *p* = 0.3; Fig. 1).

The observations from the current study confirm the prognostic validity of the current WHO criteria for defining leukemic transformation in PMF and the appropriateness, in this regard, of utilizing PB blast percentage, irrespective of BM blast content. The particular issue carries significant importance for practice because of the difficulty in obtaining adequate tissue and accurately quantifying blast content in the BM of patients with PMF, especially during accelerated and blast phase disease. Immunostaining of BM biopsy specimens for CD34 is unlikely to resolve the issue because CD34 also stains megakaryocytes, endothelial cells, and is also broadly expressed in myeloid progenitors in PMF that do not necessarily meet the morphologic criteria for blasts. Furthermore, the presence of significant bone marrow fibrosis and osteosclerosis makes it that much harder to accurately estimate BM blast count. The current study was not designed to address the issue of accelerated phase PMF and whether or not the presence of excess blasts, in otherwise chronic phase PMF, carries prognostic relevance that is independent of current prognostic models^7^.
